# Testing for population differences in evolutionary responses to pesticide pollution in brown trout (*Salmo trutta*)

**DOI:** 10.1111/eva.13132

**Published:** 2020-09-20

**Authors:** David Nusbaumer, Lucas Marques da Cunha, Claus Wedekind

**Affiliations:** ^1^ Department of Ecology & Evolution University of Lausanne Lausanne Switzerland

**Keywords:** additive genetic variance, egg size, maternal environmental effects, pesticides, salmonid, tolerance

## Abstract

Pesticides are often toxic to nontarget organisms, especially to those living in rivers that drain agricultural land. The brown trout (*Salmo trutta*) is a keystone species in many such rivers, and natural populations have hence been chronically exposed to pesticides over multiple generations. The introduction of pesticides decades ago could have induced evolutionary responses within these populations. Such a response would be predicted to reduce the toxicity over time but also deplete any additive genetic variance for the tolerance to the pesticides. If so, populations are now expected to differ in their susceptibility and in the variance for the tolerance depending on the pesticides they have been exposed to. We sampled breeders from seven natural populations that differ in their habitats and that show significant genetic differentiation. We stripped them for their gametes and produced 118 families by in vitro fertilization. We then raised 20 embryos per family singly in experimentally controlled conditions and exposed them to one of two ecologically relevant concentrations of either the herbicide S‐metolachlor or the insecticide diazinon. Both pesticides affected embryo and larval development at all concentrations. We found no statistically significant additive genetic variance for tolerance to these stressors within or between populations. Tolerance to the pesticides could also not be linked to variation in carotenoid content of the eggs. However, pesticide tolerance was linked to egg size, with smaller eggs being more tolerant to the pesticides than larger eggs. We conclude that an evolutionary response to these pesticides is currently unlikely and that (a) continuous selection in the past has either depleted genetic variance in all the populations we studied or (b) that exposure to the pesticides never induced an evolutionary response. The observed toxicity selects against large eggs that are typically spawned by larger and older females.

## 1 INTRODUCTION

Ecosystems are being altered at unprecedented rates by anthropogenic pressures that often threaten natural populations (Hendry et al., 2008). Among these threats, synthetic pesticides and their transformation products are a significant and relatively novel menace to ecosystems. Their worldwide consumption has gone through more than a 36‐fold increase between 1960 and 2005 (Zhang et al., 2011). Rivers whose catchments include agricultural and urban land receive direct and indirect pesticides influx (Chèvre, 2014; Moschet et al., 2014), and these molecules can threat aquatic organisms even at low concentrations (DeLorenzo et al., 2001; Schulz, 2004).

Moschet et al. (2014) developed a broad screening system to detect and quantify pesticides in surface waters and tested it in 5 different catchments of Switzerland. Some of the screened compounds had high occurrence and concentrations above those predicted by the Average Annual Environmental Quality Standard (AA‐EQS). Among them are S‐metolachlor, a chloroacetanilide herbicide widely used in agriculture (Zemolin et al., 2014), and diazinon, an organophosphate insecticide that had been used in urban and agricultural areas (Giddings et al., 2000) but has not been permitted in Switzerland since 2011. S‐metolachlor was detected with a frequency of 100% in all the studied catchments, exceeding its AA‐EQS (270 ng/L) on nine occasions with the highest concentration reported at 960 ng/L. Diazinon was also detected in all the studied catchments, being found in 62% of samples, exceeding the AA‐EQS (15 ng/L) in 8 samples and with the highest concentration reported at 43 ng/L (Moschet et al., 2014).

The Aare river network hosts several brown trout (*Salmo trutta*) populations that differ in their ecology, are genetically distinct and are closely monitored (Burkhardt‐Holm et al., 2002; Stelkens et al., 2012). Sexually mature trout typically spawn between October and early January (Riedl & Peter, 2013), whereas pesticide usage is most prevalent during spring and summer. One may therefore disregard this potential threat for embryos of this species. However, in their screening, Moschet et al. (2014) sampled streams from March to October and could already detect up to 30 different substances in March. For some streams, the highest pesticides concentrations were even detected in early spring. For instance, S‐metolachlor is typically applied in early spring to maize crops before germination (O'Connell et al., 1998). The incubation time of eggs in brown trout is comparatively long, temperature dependent and lasts up to half a year from oviposition to emergence (Elliott, 1994). On top of this, lipophilic pollutants tend to be adsorbed by the sediments and remain in the gravel bed in which eggs are deposited (Calvet, 1989). Also, in oviparous species, maternal transfer of contaminants is typical during oogenesis, with concentrations being four times higher in the eggs than in the female's body (Kadokami et al., 2004). Brown trout embryos can thus be expected to be chronically exposed to pesticides during their development.

There is much scientific evidence for the adverse effects that both S‐metolachlor and diazinon have on aquatic organisms. For instance, S‐metolachlor exposures were linked to significant increases in the percentage of abnormal larvae in the pacific oyster *Crassostrea gigas* (Gamain et al., 2016) and to decreases of immune system parameters in the common carp (*Cyprinus carpio*) (Dobsikova et al., 2011) and the Japanese medaka (*Oryzias latipes*) (Jin et al., 2011). Similarly, diazinon exposures have led to increased cortisol levels and promoted histopathological changes in larvae of roach (*Rutilus rutilus*) (Katuli et al., 2014) and liver tissue of rainbow trout (*Oncorhynchus mykiss*) (Banaee et al., 2013), and they have disrupted antipredator and homing behaviours of chinook salmon (*Oncorhynchus tashawytsha*) (Scholz et al., 2000). Although the toxicological effects of S‐metolachlor and diazinon have been explored in fish, the question whether natural fish populations have the potential to rapidly adapt to the detrimental effects of pollution by these pesticides is poorly explored (Klerks et al., 2011).

An evolutionary response to pesticides is typically based on standing variation and/or de novo mutations on target‐site encoding genes. De novo mutations are often responsible for the evolution of resistances in target species, especially in the case of fungicides, but also of herbicides and insecticides (Hawkins et al., 2019), because most target species have large population sizes and short generation times, and the adaptive challenge is typically very specific (e.g. one of few chemicals to react to). Nontarget vertebrate species typically have smaller population sizes and longer generation times. This is why their evolutionary responses are expected to be based mainly on standing variation (Hendry et al., 2011). One important question in evolutionary ecotoxicity is therefore whether there is additive genetic variation for tolerance to anthropogenic stressors (Bickham, 2011; Brady et al., 2017).

Various approaches have been used to demonstrate such genetic variation and even an evolutionary response to pollutants. One often used approach is sampling more and less exposed populations and testing embryos or larvae in acute toxicity test (Cothran et al., 2013; Hua et al., 2017; Whitehead et al., 2017) or using genomic analyses to test for an evolutionary response to selection (Laporte et al., 2016; Whitehead et al., 2017). This even led, in some cases, to the identification of the mechanistic basis of a specific resistance (Oziolor et al., 2019; Wirgin et al., 2011). In another approach, breeders are sampled from wild populations and used in full‐factorial in vitro breeding to produce embryos and larvae that can then be used to experimentally separate the variance components in acute toxicity tests. Using this approach, Brazzola et al. (2014) could show additive genetic variation in the tolerance to oestrogen pollution in two species of Alpine whitefish (*Coregonus* spp.), and Bridges and Semlitsch (2001) found additive genetic variation in the tolerance to the insecticide Carbaryl in southern leopard frogs (*Rana sphenocephala*).

External fertilizers with large egg numbers and no parental care such as those studied by Brazzola et al. (2014) and Bridges and Semlitsch (2001) are ideal model species to demonstrate additive genetic variance (*V*_A_) if it exists, because experimental families are easy to produce and sire effects on offspring performance are a good proxy of additive genetic effects (Lynch & Walsh, 1998). The brown trout is such an external fertilizer with no parental care. We sampled some of the natural populations that showed significant genetic differentiation in Stelkens et al. (2012) and that live in habitats under various anthropogenic influences (Marques da Cunha et al., 2019) to test whether environmentally relevant concentrations of S‐metolachlor and diazinon can affect fitness‐related traits of brown trout embryos, and whether populations differ in their potential to evolve in response to pollution by these pesticides.

## 2 METHODS

### 2.1 Fish sampling and handling

At the beginning of the breeding season (i.e. during October), adult brown trout were caught from seven different locations (“populations”) within a stream network of the river Aare by electrofishing and kept in the *Fischereistützpunkt* Reutigen (the cantonal hatchery). From then on, females were palpated for signs of ovulation in intervals of 7 days. If final egg maturation and ovulation had happened, eggs were stripped by hand and used for the routine hatchery breeding with freshly stripped milt from males of the same population as the females (within‐population crossings). The experimental crossings for the present study were all done at one day (December 16) towards the end of the spawning season. On that day, 61 males and 59 females were narcotized with 0.075 g/L Tricaine‐S (MS‐222) buffered with 0.15 g/L NaHCO_3_, weighted and photographed on each side under standardized condition (focal length 17 mm, 1/400, f/4.0, ISO 400, white balance 4,000 K) in a custom‐made photograph box and stripped for their gametes. These gametes were used for in vitro fertilizations.

**Figure 1:**
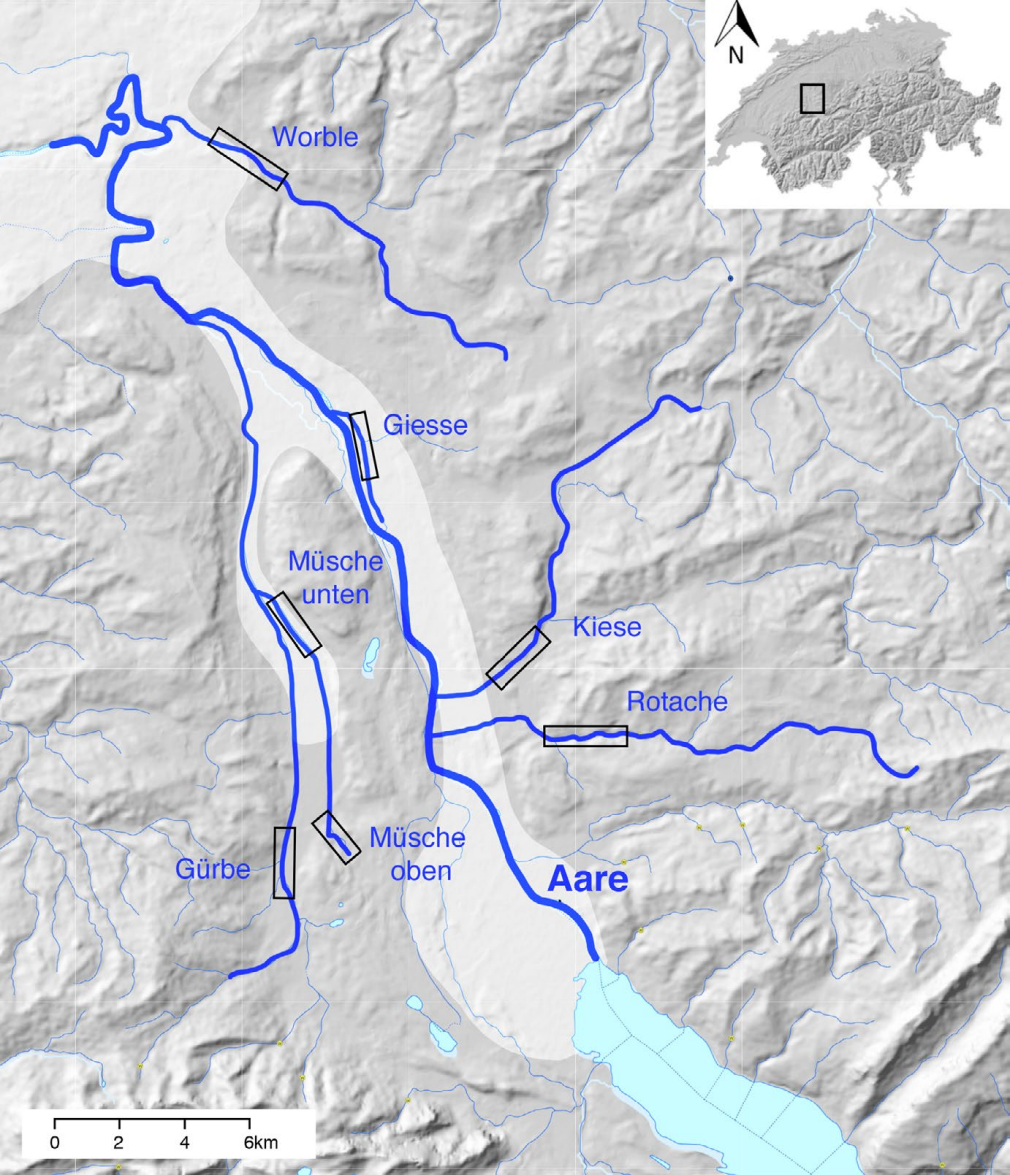
Map of the study area illustrating the origins of the samples (rectangles) within the streams that were sampled. The main river Aare (thickest stream line) flows North and was not sampled. The map of Switzerland in the upper right corner indicates the location of the map section. This figure was generated on the Swiss Federal Office for Topography (https://map.geo.admin.ch), combining the surface elevation map, hydrology and the climate suitability for agriculture. The area for which climate suitability is considered “very favourable for arable farming” is shaded in light grey

The seven different populations we sampled (*Giesse, Gürbe, Kiese, Müsche unten, Müsche oben, Rotache* and *Worble*; Figure 1) showed significant genetic and phenotypic differentiation in earlier studies (Pompini et al., 2013; Stelkens et al., 2012). The locations differ in topographic slope gradients (Stelkens et al., 2012) and land use (see Figure 1). The locations *Giesse* and *Müsche unten* represent typical slow‐flowing streams in areas that are mainly used by agriculture and horticulture, the locations *Rotache* and *Müsche oben* represent faster flowing streams in largely unpopulated areas that are dominated by forests and pasture, and the locations *Gürbe* and *Worble* represent more populated areas (small villages with a few thousand inhabitants) with some pasture, agriculture and forests.

### 2.2 Gametes collection and fertilization

Females were stripped into plastic containers while males were stripped into Petri dishes, carefully avoiding contamination by faeces, urine or water. Males and females were split into 28 (2 × 2) and 3 (1 × 2) breeding blocks each consisting of 2 or 1 females crossed with 2 males of the same population, resulting in a total of 118 different sib groups. Fertilizations were all done at the same day and as described by Jacob et al. (2010). Briefly, eggs were fertilized by family in Petri dishes through activation of the sperm with standardized water (OECD, 1992) and left for 2 hr undisturbed to harden. Twenty‐four embryos of each sib group were then transferred to 50 ml Falcon tubes filled with standardized water and transported on ice to the laboratory. There, eggs were washed under tap water as described by von Siebenthal et al. (2009) and distributed to 24‐well plates (Falcon) filled with 1.8 ml of autoclaved standardized water per well in a climate chamber at 6.9°C (one embryo per well). Four embryos per sib group were used in another study on possible interaction effects of pesticides. The sample size for the present study is therefore 20 eggs per family = 2,360 embryos in total.

#### 2.2.1 Treatment and offspring performance

One day after fertilizations, embryos were exposed to a low or a high concentration of S‐metolachlor or diazinon, or to a control (i.e. autoclaved standardized water), by adding 0.2 ml of the corresponding spike solution to each well. Both chemicals were obtained from Sigma‐Aldrich, Switzerland. We aimed at concentrations of 0.25 and 1 µg/L for S‐metolachlor and of 15 and 45 ng/L for diazinon, with the lower and higher concentrations approximating the AA‐EQS and the highest reported concentration in Swiss streams by Moschet et al. (2014) of each compound, respectively. Later quantification of S‐metolachlor and diazinon (see below) in spike water solution turned out to be lower than that. For diazinon, spike solutions for the low and high concentrations were 112 and 269.8 ng/L, respectively. Embryos were therefore exposed to 11.2 and 26.98 ng/L of diazinon. The actual concentrations of S‐metolachlor to which the embryos were exposed were 65 and 252 ng/L.

Embryos were monitored to record mortality and timing of hatching, with no water exchange until hatching that happened 65 to 74 days after fertilization (mean ± *SD* = 69.0 ± 1.8). On the day of hatching, embryos were singly transferred to 12‐well plates (Falcon) filled with 3 ml of autoclaved standardized water per well, that is exposure to the pesticides stopped by then (apart from the contamination that could not be avoided during the transfer of the larvae). These plates were then photographed from below (to avoid water‐induced image distortion) in a custom‐made photograph box for body measurements on the larvae. After 14 days, these plates were again photographed for measurement of larval standard length (from the tip of the nose to the end of the end of the last vertebra, that is the base of the tail, without the caudal fin) and yolk sac volume (calculated as in Jensen et al. (2008)) at hatching and 14 days later to determine larval growth (difference of standard length between the time points) and yolk sac consumption (difference of volume between the time points).

### 2.3 Quantitative analysis of S‐metolachlor and diazinon

The quantification of pesticides in spike solution was done by the Central Environmental Laboratory from the Environmental Engineering Institute, EPFL, Switzerland. To quantify the actual concentration in the spike solutions, an UPLC®‐ON‐LINE‐SPE‐MS/MS (Acquity Xevo‐TQ; Waters Corporation) was used. The samples were spiked with a standard mixture of surrogate, containing diazinon d10 and S‐metolachlor d6 and filtered with glass fibre filter (Simplepure PP + GF, 0.45 µm, 25 mm, BGB). Five standard solutions (range 10 to 350 ng/L) have followed the same preparation. Five mL of each sample was loaded on an SPE column (Oasis HLB 25 µm, 2.1 × 20 mm, Waters) with ultrapure water, acidified at 1% of formic acid, as eluent. The online transfer of compounds to the analytical column (Acquity HSS T3, 1.8 µm, 2.1 × 100 mm, Waters) was made with a gradient of ultrapure water and acetonitrile acidified at 0.1% of formic acid. Multiple reaction monitoring modes with two transitions were used to detect the compounds, and the quantification was performed with internal standard calibration.

### 2.4 Measurement of breeder size, egg volume and egg colour

Breeder standard lengths were measured from standardized photographs in Fiji (Schindelin et al., 2012). Egg size and clutch size of each female were inferred from standardized pictures of the Petri dishes of sib groups, taken 2 hr after fertilization on a LED light board within a closed polystyrene box to control for light conditions. A macro was designed in Fiji to first extract the number of eggs per sib group. This number was determined using two different levels of thresholding (to detect both pale and coloured eggs), and the highest count value was retained. Then, a sub‐sample of 196.5 ± 1.6 (± *SE*) eggs each that were not clogged together in the picture were analysed with another macro for egg characteristics. With this other macro, the section surface (detected through particle analysis, that is a function in Fiji that allows detecting particles of given characteristics) and the mean redness and yellowness (a* and b* components of the CIE‐Lab colour space, respectively) of these sections were determined for each egg. Egg volume (in mm^3^) was inferred from section surface. Eggs volumes, a* and b* were averaged for each female. Egg redness and yellowness were used as a proxy of carotenoid content, that is mostly astaxanthin, zeaxanthin and lutein that have previously been found in our study populations and that support embryo development (Wilkins et al., 2017; Wilkins, et al., 2017). Astaxanthin and zeaxanthin both affect egg redness (Wedekind et al., 1998; Wilkins, et al., 2017), while lutein affects yellowness (Wedekind et al., 1998).

### 2.5 Statistical analyses

Data resulting from exposure to the two different pesticides were analysed separately. In each case, mixed‐effects models were used from the *lme4* package (Bates et al., 2015) with sire, dam and population as random effects, and pesticide exposure (“e”; yes/no) and pesticide concentration (“c”; low/high) as fixed effects. Analogous models that combine pesticide exposure and concentration to a factor “treatment” (“t”; control, low, high) lead to similar conclusions (Supplementary Table S1).

Mortality was analysed using generalized linear mixed‐effects model with a binomial error distribution, while all other response variables were analysed using linear mixed‐effects models. For the analysis of growth, hatchling length was entered as a further fixed effect. Likewise, hatching time was included as a further fixed effect for the analysis of yolk sac volume at hatching, and the latter was entered as a fixed effect for the analysis of yolk consumption. Models were fitted using maximum likelihood. The significance of a term in each model was tested by adding or removing it and testing this model against a reference model. Model fits were compared with likelihood ratio tests (LRT) and Akaike information criterion (AIC). Linear models were used to explain female reaction norms to the pesticides (i.e. ß coefficients from models containing a significant e × d interaction term, i.e. significant variation in female tolerance) with egg traits as predictors (i.e. egg volume, egg yellowness and egg redness). Paternal reaction norms were calculated analogously, from models containing e × s interactions.

Analyses of variance were used to test variation in breeder's length and egg traits among population. The relationship between female size, clutch size and egg size was tested with LRT on linear mixed‐effects model in which population was entered as a random factor and female size as a fixed effect. Extraction of variance components was done for each treatment using *VarCorr* from the *lme4* package on random‐intercept from mixed effect models fitted with dam, sire, dam × sire, block and population as random effects. Additive genetic variance (*V*_A_) was calculated as four times the sire component, dominance variance (*V*_D_) as four times the dam × sire component, narrow‐sense heritability (*h* ^2^) as *V*_A_ divided by the total variance, mean‐scaled additive genetic variance (*I*_A_) as *V*_A_ divided by the square mean trait value and coefficients of additive genetic variation (CV_A_) as the square‐root of *V*_A_ divided by the trait mean, multiplied by one hundred (Hansen et al., 2011; Houle, 1992; Lynch & Walsh, 1998). This was done for all embryo, and larval characteristics that were measured except for the dichotomous variable embryo mortality because overall mortality turned out too low for estimating the variance components. All the statistical analyses and the calculations of the variance components were performed in Rstudio (RStudioTeam, 2015).

## 3 RESULTS

### 3.1 Effects of S‐metolachlor on offspring performance

**Figure 2:**
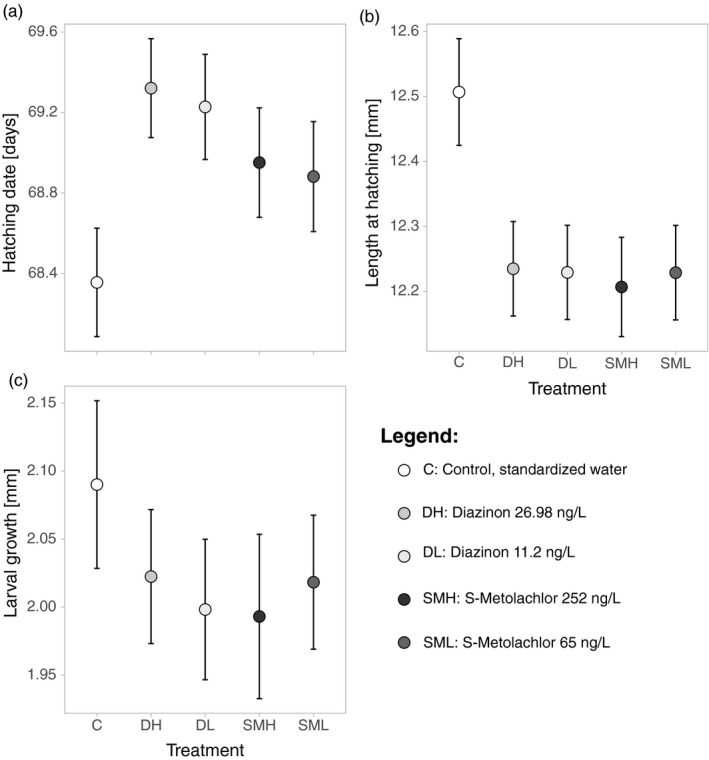
Effects of exposure to two concentrations of S‐metolachlor and diazinon on (A) hatching time, (B) length at hatching and (C) larval growth during 14 days after hatching. Plot shows means based on family means and 95% CI. See Table 1 for statistics

There was no significant effect of S‐metolachlor on embryo survival (Table 1A). Mortality was low and similar between the controls (2.3 ± 2.2%; mean ± 95% CI), the embryos exposed to the low concentration (2.0 ± 1.9%) and the high concentration (1.7 ± 1.3%) of S‐metolachlor (Supplementary Figure S1a). Hatching time was delayed by the pesticide exposure but there was no significant difference between the two concentrations tested (Table 1B, Figure 2a). Larvae exposed to the pesticide hatched smaller and grew slower than their siblings. Again, the concentration did not play a significant role here (Table 1C,D, Figure 2b,c). The size of their yolk sac at hatching and yolk consumption afterwards were not significantly affected by the pesticide treatment, nor by its concentration (Table 1E,F; Figure S1b,c).

**Table 1:** Likelihood ratio tests on generalized linear mixed‐effects models (GLMM) on mortality (A), linear mixed‐effects models (LMM) on hatching time (B), larval length at hatching (C), larval growth during 14 days after hatching (D), yolk sac volume at hatching (E) and yolk consumption during 14 days after hatching (F)

Model terms	Effect tested	AIC	*df*	χ^2^	P	AIC	*df*	χ^2^	P
		S‐metolachlor				Diazinon			
(A) Mortality									
c + e + p + s + d		194	6			177	6		
c + p + s + d	e	192	5	<0.1	0.89	175	5	0.2	0.68
e + p + s + d	c	193	5	0.4	0.54	175	5	<0.1	0.88
c + e + s + d	p	192	5	0	1	175	5	0	1
c + e + p + d	s	192	5	0	0.99	176	5	1.4	0.24
c + e + p + s	d	211	5	18.7	**<0.001**	186	5	11.1	**<0.001**
c + e + e|p + s + d	e × p	198	8	0.6	0.75	181	8	0	1
c + e + p + e|s + d	e × s	198	8	0.1	0.97	179	8	2.1	0.35
c + e + p + s + e|d	e × d	192	8	6.5	**0.04**	177	8	3.7	0.16
(B) Hatching time									
c + e + p + s + d		4,288	7			4,391	7		
c + p + s + d	e	4,301	6	14.6	**<0.001**	4,422	6	33.3	**<0.001**
e + p + s + d	c	4,289	6	3.1	0.08	4,390	6	0.9	0.34
c + e + s + d	p	4,287	6	1.3	0.26	4,390	6	1.6	0.21
c + e + p + d	s	4,331	6	45.2	**<0.001**	4,440	6	51.1	**<0.001**
c + e + p + s	d	4,486	6	199.6	**<0.001**	4,540	6	151.1	**<0.001**
c + e + e|p + s + d	e × p	4,291	9	0.7	0.72	4,394	9	1.3	0.52
c + e + p + e|s + d	e × s	4,291	9	0.9	0.63	4,392	9	2.8	0.25
c + e + p + s + e|d	e × d	4,292	9	0.2	0.91	4,393	9	1.6	0.45
(C) Length at hatching									
c + e + p + s + d		599	7			741	7		
c + p + s + d	e	682	6	85.3	**<0.001**	803	6	64.4	**<0.001**
e + p + s + d	c	599	6	1.8	0.19	740	6	0.7	0.39
c + e + s + d	p	599	6	0.8	0.37	742	6	2.4	0.12
c + e + p + d	s	615	7	18.0	**<0.001**	758	6	18.9	**<0.001**
c + e + p + s	d	865	7	268.0	**<0.001**	931	6	192.0	**<0.001**
c + e + e|p + s + d	e × p	602	9	1.4	0.51	745	9	0.2	0.91
c + e + p + e|s + d	e × s	600	9	3.1	0.22	745	9	0	0.99
c + e + p + s + e|d	e × d	595	9	8.2	**0.02**	733	9	11.9	**0.002**
(D) Growth									
l + c + e + p + s + d		606	8			728	8		
l + c + p + s + d	e	609	7	4.9	**0.03**	732	7	6.5	**0.01**
l + e + p + s + d	c	606	7	2.3	0.13	726	7	0	0.85
c + e + p + s + d	l	635	7	31.3	**<0.001**	774	7	48.2	**<0.001**
l + c + e + s + d	p	604	7	0	1	726	7	0	1
l + c + e + p + d	s	606	7	1.9	0.17	728	7	2	0.15
l + c + e + p + s	d	631	7	27.1	**<0.001**	754	7	28.5	**<0.001**
l + c + e + e|p + s + d	e × p	610	10	0.2	0.91	731	10	0.6	0.73
l + c + e + p + e|s + d	e × s	609	10	1.2	0.54	731	10	1.2	0.56
l + c + e + p + s + e|d	e × d	609	10	1.3	0.51	731	10	0.4	0.83
(E) Yolk volume at hatching									
h + c + e + p + s + d		6,725	8			6,892	8		
h + c + p + s + d	e	6,703	7	0.9	0.35	6,890	7	0.1	0.67
h + e + p + s + d	c	6,724	7	1.8	0.18	6,890	7	0.3	0.57
c + e + p + s + d	h	6,735	7	12.2	**<0.001**	6,912	7	22.0	**<0.001**
h + c + e + s + d	p	6,737	7	13.9	**<0.001**	6,902	7	12.5	**<0.001**
h + c + e + p + d	s	6,723	7	0	1	6,901	7	11.5	**<0.001**
h + c + e + p + s	d	6,758	7	35.4	**<0.001**	6,903	7	13.8	**<0.001**
h + c + e + e|p + s + d	e × p	6,727	10	1.7	0.43	6,893	10	2.24	0.33
h + c + e + p + e|s + d	e × s	6,729	10	0	1	6,896	10	0	1
h + c + e + p + s + e|d	e × d	6,724	10	4.4	0.11	6,892	10	13.1	**0.001**
(F) Yolk consumption									
g + v + c + e + p + s + d		3,443	9			3,545	9		
g + v + c + p + s + d	e	3,444	8	2.8	0.09	3,546	8	3.4	0.06
g + v + e + p + s + d	c	3,441	8	0.1	0.78	3,543	8	0.1	0.80
g + c + e + p + s + d	v	4,001	8	559.7	**<0.001**	4,130	8	587.8	**<0.001**
v + c + e + p + s + d	g	3,443	8	1.7	0.19	3,544	8	1.2	0.27
g + v + c + e + s + d	p	3,441	8	0	1	3,542	8	0	1
g + v + c + e + p + d	s	3,444	8	2.6	0.11	3,542	8	<0.1	0.95
g + v + c + e + p + s	d	3,478	8	36.6	**<0.001**	3,576	8	33.2	**<0.001**
g + v + c + e + e|p + s + d	e × p	3,447	11	0	1	3,549	11	0	1
g + v + c + e + p + e|s + d	e × s	3,447	11	<0.01	0.99	3,548	11	0.8	0.66
g + v + c + e + p + s + e|d	e × d	3,446	11	1	0.61	3,546	11	3.0	0.23

Note

Fixed effects: e, exposure (yes/no); c, concentration (low/high); h, hatching time; l, length at hatching; v, yolk volume at hatching; g, growth. Random effects: p, population; s, sire; d, dam. Models including or lacking the term of interest were compared with the reference model to determine the significance of the effect tested. Significant p‐values are highlighted in bold.

### 3.2 Effect of diazinon on offspring performance

There was no significant effect of diazinon on embryo survival (Table 1A). Mortality was low and similar between the controls (2.3 ± 2.2%), the embryos exposed to the low concentration (1.6 ± 1.6%) and the high concentration (1.4 ± 1.4%) of diazinon (Figure S1a). Hatching time was delayed by the pesticide exposure regardless of its concentration (Table 1B, Figure 2a). Larvae exposed to the pesticide hatched at smaller size and grew slower afterwards than their siblings, but the concentration did not play a significant role (Table 1C,D, Figure 2b,c). The size of their yolk sac at hatching and yolk consumption were not significantly affected by the pesticide treatment, nor by its concentration (Table 1E,F; Figure S1b,c).

### 3.3 Population differences in breeder and egg characteristics

Breeder body length varied among populations, and males were on average larger than females (two‐way ANOVA, effect of population: *F*_2,6_ = 21.5, *p* < .001, effects of sex: *F*_2,1_ = 22.5, *p* < .001). When controlling for potential population effects on egg size, female body length was positively correlated to clutch size (LRT: χ^2^ = 38.5, *p* < .001; Figure S2a) and mean egg size (LRT: χ^2^ = 4.6, *p* = .03; Figure S2b). Populations differed in mean egg yellowness (*F*_1,6_ = 2.5, *p* = .03; Figure S3a) and mean egg redness (*F*_1,6_ = 3.3, *p* = .007; Figure S3b).

### 3.4 Population and parental effects on tolerance to S‐metolachlor and diazinon

There were no significant population‐specific responses to the treatments (i.e. no significant e × p interaction terms in Table 1). There were also no other significant population differences in offspring traits except for yolk sac volume at hatching (Table 1E).

**Figure 3:**
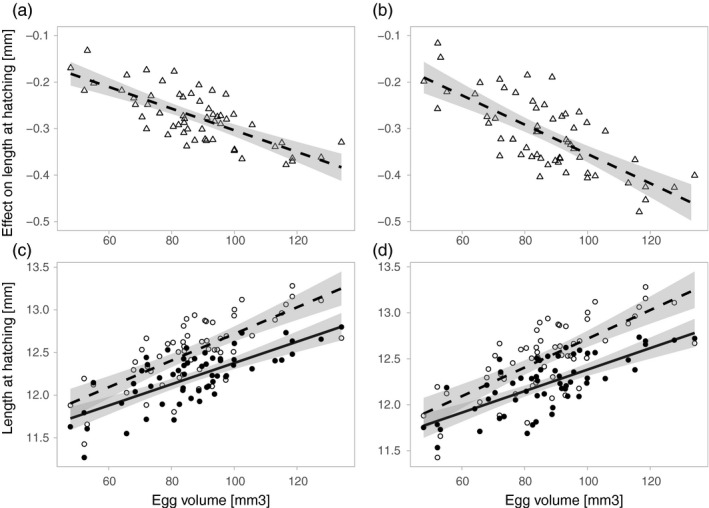
Relationship between egg volume and effects of S‐metolachlor (A&C) or diazinon (B&D) on larval length at hatching. In panels A and B, triangles show treatment effect for each dam, that is ß‐coefficients extracted from the linear mixed effect model containing the interaction term e × d (see Table 1). Panels C and D show the relationship between mean hatchling's length per dam and egg volume when exposed to the control (dashed line and open circles) or the pesticide (solid line and filled circles). Linear regressions are drawn with 95% confidence interval. See Table 2 for statistics

Dam effects were significant for all embryo traits that were determined (Table 1A–F). Significant dam effects on tolerance to pesticides (e × d interactions) were found for mortality induced by S‐metolachlor (Table 1A), for length at hatching in response to both pesticide (Table 1C), and for yolk volume at hatching affected by diazinon (Table 1E). This variation in female tolerance was significantly associated with mean egg volume for length at hatching, but not for mortality and yolk volume at hatching (Table 2). Egg colour did not explain variance in female tolerance to both pesticides (Table 2). Females producing larger eggs were more severely affected by the treatment than those producing smaller eggs, both in absolute (Table 2, Figure 3) and in relative hatchling length (Figure S4). Variance in tolerance among females was not found in larval growth nor yolk consumption (Table 1D,F).

**Table 2:** Summary of linear models on the effect of egg traits on female individual response to S‐metolachlor (A & B) and diazinon (C & D) when considering mortality (A), larval length at hatching (B & C) and yolk volume at hatching (D)

Coefficients	Estimate	S.E.	t	*P*
(A) Mortality: S‐metolachlor				
(Intercept)	−1.656	4.05	−0.41	0.68
Egg volume	0.033	0.02	1.78	0.08
Egg redness	−0.053	0.04	−1.40	0.17
Egg yellowness	0.010	0.07	0.15	0.88
(B) Length at hatching: S‐metolachlor				
(Intercept)	0.024	0.06	0.37	0.71
Egg volume	−0.002	0.00	−7.74	**<0.001**
Egg redness	0.000	0.00	−0.80	0.43
Egg yellowness	−0.001	0.00	−1.34	0.19
(C) Length at hatching: diazinon				
(Intercept)	0.087	0.09	1.00	0.32
Egg volume	−0.003	0.00	−7.22	**<0.001**
Egg redness	0.000	0.00	−0.36	0.72
Egg yellowness	−0.002	0.00	−1.59	0.12
(D) Yolk volume at hatching: diazinon				
(Intercept)	−1.637	4.79	−0.34	0.73
Egg volume	0.039	0.02	1.77	0.08
Egg redness	0.013	0.04	0.29	0.78
Egg yellowness	−0.041	0.08	−0.53	0.60

Note

Models *F* tests (*df* =always 55): A) *r* ^2^ = 0.03, *F* = 1.5, *p* = .21, B) *r* ^2^ = 0.57, *F* = 26.5, ***p* < .001**, C) *r* ^2^ = 0.54, *F* = 23.3, ***p* < .001** D) *r* ^2^ = <0.01, *F* = 1.1, *p* = .35. Female individual response has been extracted from the coefficients estimates of the mixed‐effects models containing the interaction term e × d (see Table 1). Significant p‐values are highlighted in bold.

Sires’ identities had no effect on offspring mortality (Table 1A) but significantly explained variation in hatching traits (i.e. timing, length and yolk volume, see Table 1B,C,E). However, we did not find evidence for additive genetic variance in any offspring traits (i.e. no significant e × s interaction, see Table 1; Supplementary Figure S5). Table 3 lists the variance components of the phenotypic variation in the controls and the two types of pesticide exposure, the narrow‐sense heritabilities (*h* ^2^), and mean‐scaled additive genetic variance (*I*_A_), and the coefficients od variation (CV_A_) (see Supplementary Table S2 for the analogous table for exposure to low and high concentration of each pesticide).

**Table 3:** Maximum likelihood estimates of variance components for (a) hatching time, (b) length at hatching, (c) larval growth, (d) yolk sac volume at hatching and (e) yolk consumption during the first 14 days after hatching

	*V*_Tot_	*V*_A_	*V*_Dam_	*V*_D_	*V*_Block_	*V*_Pop_	*V*_Res_	*h* ^2^	*I_A_*	CV_A_
(A) Hatching time											
Control	3.33	0.17	0.40	<0.01	1.22	0.05	1.62	0.05	<0.0001	0.60
S‐Metolachlor	3.34	0.83	0.53	0.48	0.92	<0.01	1.56	0.25	0.0002	1.32
Diazinon	3.32	1.13	0.41	0.50	0.76	<0.01	1.74	0.34	0.0002	1.54
(B) Length at hatching											
Control	0.22	0.02	0.11	0.02	<0.01	0.01	0.08	0.07	0.0001	0.98
S‐metolachlor	0.18	0.03	0.06	0.07	0.01	<0.01	0.08	0.14	0.0002	1.33
Diazinon	0.20	<0.01	0.04	0.13	0.01	0.01	0.10	<0.01	<0.0001	<0.01
(C) Growth											
Control	0.15	0.03	0.01	<0.01	<0.01	<0.01	0.13	0.18	0.0063	7.95
S‐metolachlor	0.13	<0.01	<0.01	0.04	<0.01	<0.01	0.11	<0.01	<0.0001	<0.01
Diazinon	0.16	0.02	0.01	0.01	<0.01	<0.01	0.14	0.16	0.0061	7.82
(D) Yolk sac volume at hatching											
Control	132.14	<0.01	8.73	<0.01	11.50	11.08	100.83	<0.01	<0.0001	<0.01
S‐metolachlor	134.06	<0.01	16.60	<0.01	11.25	7.29	98.92	<0.01	<0.0001	<0.01
Diazinon	135.09	26.05	13.13	7.20	12.17	5.95	95.53	0.19	0.0147	12.13
(E) Yolk consumption											
Control	95.76	<0.01	7.90	<0.01	11.15	9.85	66.87	<0.01	<0.0001	<0.01
S‐metolachlor	96.74	<0.01	18.57	10.59	3.11	2.99	69.42	<0.01	<0.0001	<0.01
Diazinon	87.55	12.18	7.46	9.98	2.51	2.21	69.82	0.14	0.0431	20.75

Note

Based on these estimates, narrow‐sense heritability (*h^2^*), mean‐scaled additive genetic variance (*I_A_*) and coefficients of additive genetic variation (CV_A_) were calculated for each of type of exposure.

Abbreviations: *V*_A_, additive genetic; *V*_Block_: block; *V*_D_, dominance; *V*_Dam_, maternal; *V*_Pop_: population; *V*_Res_, residual; *V*_Tot_, total variance.

## 4 DISCUSSION

We experimentally tested whether exposure to ecologically relevant doses of S‐metolachlor or diazinon affected brown trout during embryogenesis and early larval growth. We studied seven populations that differ in habitats and in exposure to pesticides, and we tested for additive genetic variance in pesticide tolerances within and between the populations. We also tested for maternal environmental effects on pesticide tolerances and explored possible links to egg characteristics. We found that both pesticides affected embryo and larval growth at all concentrations. There were no significant population differences in these toxic effects and no statistically significant additive genetic variance for a tolerance to the pesticides. However, there were strong maternal effects on pesticide tolerance that could be linked to egg size (but not to carotenoid contents).

None of the two pesticides induced significant mortality at the ecologically relevant concentrations that we tested them. However, S‐metolachlor and diazinon both slowed down embryonic and larval development at all concentrations. Embryos exposed to these pesticides hatched later, at smaller size, and the hatchling grew slower during the first 14 days after hatching.

The responses we observed in reaction to the herbicide S‐metolachlor confirm previous findings in Perez's frog (*Pelophylax perezi*) and zebrafish (*Dario rerio*) embryos (Quintaneiro et al., 2017, 2018). S‐metolachlor is a chloroacetamide and targets biosynthesis of fatty acid (DeLorenzo et al., 2001), thus inhibiting mitosis and cell division (Quintaneiro et al., 2017). In fish, it can interfere with steroidogenesis and impair biochemical pathways involved in neurotransmission and energy production (Quintaneiro et al., 2017). The impairment of processes involved in cell division, and energy production could therefore be responsible for the effects we observed. Quintaneiro et al. (2017) suggested that delays in hatching time may be caused by disfunction of the hatching hormone chorionase.

The responses we observed in reaction to diazinon also confirm previous findings on zebrafish and medaka (Cao et al., 2018; Flynn et al., 2018). Diazinon is an organophosphate insecticide that inhibits acetylcholinesterase (Pope, 1999; PPDB, 2019) and is thus neurotoxic in target and many nontarget species (Pope, 1999). It can also lead to mitochondrial dysfunction and affect oxidative respiration and ATP synthesis, which could explain the observed reduction in developmental rate (Cao et al., 2018; Karami‐Mohajeri & Abdollahi, 2013).

Reduced embryo and larval development rate are expected to affect, in the wild, timing and size at emergence from the gravel bed. Larvae that emerge early and at a large size typically have a competitive advantage when establishing and defending a feeding territory and suffer less from various sources of mortality (Einum & Fleming, 2000; Good et al., 2001; Skoglund et al., 2011). The observed effects of the sublethal concentration of pesticides are therefore likely to reduce fitness.

Previous studies on S‐metolachlor and diazinon (Cao et al., 2018; Flynn et al., 2018; Quintaneiro et al., 2017) were carried out at higher concentrations than we used here, and we even found significant toxicity at concentrations below the AA‐EQS. This could be due to two reasons. First, monitoring large numbers of embryos individually provides the necessary statistical power to detect effects even if they are small. Second, with our experimental breeding we could control statistically for parental effects and thereby further increase the statistical power for toxicity testing. Marques da Cunha et al. (2019) used a similar approach and could unveil significant toxicity of only 2 pg ethinylestradiol (EE2) on brown trout embryos despite very small effect sizes. Third, embryos are known to be more susceptible to chemical pollutants than juveniles or adult stages (Mohammed, 2013), which is one of the reasons why zebrafish embryos have become an important model in ecotoxicology (OECD, 2013).

The populations we sampled differ in various habitat characteristics and show significant genetic differentiation (Stelkens et al., 2012). Adults of these populations differ in morphometry (Stelkens et al., 2012), and eggs and larvae differ in their reaction to water‐born pathogen cues (Pompini et al., 2013). However, they do not seem to differ in their reaction to important micropollutants. We did not find any population difference in reaction to the pesticides (i.e. e × p interactions in our models), and Marques da Cunha et al. (2019) could not find any population differences in the reaction to EE2. This could be due to (a) high sensitivity to concentrations below the lowest concentrations in the study area (i.e. variation among populations in the concentration of the pollutants has no significant effects because all concentrations induce selection), (b) insufficient genetic variation between populations at loci that are important for tolerance against these chemical stressors or (c) possible stage‐specific population effects, that is the possibility that population effects can only be seen at certain life‐history stages. In medaka, for example, larvae and embryos react differently to exposure to diazinon (Hamm et al., 2001).

The males used in this study varied in their genetic quality as revealed by the significant sire effects on embryos performance. Some males were of higher genetic quality than others, confirming previous observations in other brown trout populations within Switzerland (Jacob et al., 2010; Wedekind et al., 2008). However, the paternal sib groups did not vary in their response to the stressors, that is we did not find significant additive genetic variance for tolerance to either S‐metolachlor or diazinon. This kind of genetic variance would be required for populations to evolve in reaction to these pesticides.

A lack of observing genetic variance for a specific phenotype can be due to, for example, insufficient statistical power (i.e. a type II error) or to a lack of overall genetic diversity in the studied populations. With regard to the first possibility, we sampled a large number of breeders (*n* = 120) and raised 2,360 of their offspring singly in a set‐up that was previously shown to reveal small effect sizes in brown trout (Clark et al., 2013, 2016; Marques da Cunha et al., 2019). We could then detect small treatment effects on other traits (e.g. a ~2% treatment effect on larval length). A type II error is therefore unlikely. With regard to the second possibility, there is much genetic diversity within and between the populations we sampled (Stelkens et al., 2012) and no evidence for inbreeding depression as tested in population crosses (Clark et al., 2013; Stelkens et al., 2014). Moreover, we sampled males that showed significant *V*_A_ for most of the traits observed. This suggests that there is no significant lack of overall genetic diversity in our study populations.

The observed lack of genetic variation in pesticide tolerance is therefore likely due to reduced allelic diversity at loci that are important for the development of such tolerances. Genetic variance at these loci could have been depleted through selection over recent generations or may have never been present in the studied populations (Van Straalen & Timmermans, 2002). When Marques da Cunha et al. (2019) found no *V*_A_ for tolerance to EE2, they argued that this was likely an effect of rapid evolution induced by continuous selection since the market launch of the contraceptive pill, because other salmonid populations that were less exposed to this pollutant showed significant *V*_A_ for tolerance to EE2 (Brazzola et al., 2014). We know of no other studies that have searched for *V*_A_ in the tolerance to either S‐metolachlor or diazinon in salmonids or any other fish, that is we cannot offer analogous comparisons between salmonid species. It is possible that the market launch of the various pesticides has induced rapid evolution, which could have led to an increased mean tolerance to the pesticides over the generations and may thereby have depleted the *V*_A_ for this tolerance. However, further studies are necessary to test this hypothesis.

We found significant maternal effects on all the embryo and larval traits that we measured, which is typical in salmonids and mostly linked to variation in maternal investment into eggs (e.g. Wilkins et al. (2016), Wilkins, et al. (2017). Interestingly, we also found significant maternal effects in the reaction to the treatments (the significant e × d interactions in our models). These maternal effects could not be linked to egg colours that are indicators of carotenoid content, but they could be predicted by egg size. Embryos of larger eggs experienced a larger reduction in size (in absolute and relative terms) when exposed to the pesticides than embryos of smaller eggs. Embryos of larger eggs hence reacted more strongly to the treatment, that is they were more affected by the induced stress than embryos in small eggs. This finding goes against the general trend that larger eggs produce larvae that have a significant advantage over smaller ones (Pakkasmaa et al., 2001). We can offer two hypotheses to potentially explain the higher susceptibility of larger eggs. First, we found larger females to produce larger eggs, confirming previous findings in this species (Ojanguren et al., 1996), and there could well be other systematic differences between females that affect their mean egg size. It is therefore possible that large eggs differ not only in size from small eggs but also in composition (that may reveal female life history) and embryo genetics (linked, for example, to size‐related genetic differences among females or to female age). Second, larger eggs have a greater membrane surface area in contact with their environment and thus a greater adsorption capacity and may even be able to store more pesticides (because of their larger volume). This may be especially pertinent if eggs are exposed to lipophilic molecules such as S‐metolachlor and diazinon that are known to be well adsorbed by aquatic organisms (Huckins et al., 1990; Soedergren, 1987). It is therefore possible that larger eggs absorb more pesticide during embryo incubation. However, if toxicants are integrally absorbed by eggs (such as EE2 in Marques da Cunha et al. (2019), one would expect larger eggs to have an advantage because toxicants would be diluted in a larger volume. The absorption rate and intra‐egg concentrations would therefore still have to be tested. Moreover, even if larger eggs suffer more from exposure to pesticides than small eggs, they still result in larger offspring that may profit from the size‐related advantages mentioned above.

Carotenoids are important antioxidant molecules that have been shown to help brown trout embryo coping with stress (Marques da Cunha et al., 2018; Wilkins, et al., 2017). Wilkins, et al. (2017) found that carotenoid content was not linked to egg weight, that is egg size effects are likely independent of potential effects of carotenoid content. Because they also established that eggs colours are useful indicators of carotenoid content in brown trout, we could use them as proxy for carotenoid content. We found no link between egg colours and pesticide tolerances. This suggests that carotenoids do not provide significant protection against the toxic effects of important herbicides and insecticides.

In conclusion, we found significant toxicity of ecologically relevant concentrations of S‐metolachlor and diazinon in brown trout, a keystone species in many freshwater habitats. We found no additive genetic variance for tolerance to these pesticides in seven different populations. This suggests that the populations have either lost the potential to further evolve tolerance against these chemical pollutants or that they never had that potential. Large eggs that are typically spawned by large females suffer more from the pollutants than small eggs. Chemical pollution by pesticides therefore seems to select against the offspring of larger and older females and against female life‐history strategies that result in large eggs.

## 5 ETHICS

The sampling of adults, the stripping, the experimental breeding and the raising of embryos were approved by the Fishery Inspectorate of the Bern canton and by the Veterinary Office of the Bern canton (approval number BE188/14).

## CONFLICT OF INTEREST

None declared.

## AUTHOR CONTRIBUTIONS

All authors designed the experiment and organized the field work. CW did the in vitro fertilizations. DN determined egg size. DN and LMC distributed the eggs to 24‐well plates, treated and monitored the embryos and determined larval growth. All authors analysed the data and wrote the manuscript.

## Supporting information

Supplementary Material

## Data Availability

The data used in this study have been deposited on the Dryad repository https://doi.org/10.5061/dryad.j9kd51c9h.
