# Supplementary material for: Testing for population differences in evolutionary responses to pesticide pollution in brown trout (Salmo trutta)
Source: Evol Appl. 2020 Sep 20;14(2):462–75. doi: 10.1111/eva.13132 (PMC7896705; doi:10.1111/eva.13132)
Supplement: Supplementary file 1 — Supplementary Material [file EVA-14-462-s001.docx]

**Supplementary information**

**Testing for population differences in evolutionary responses to pesticide pollution in brown trout (*Salmo trutta*)**

David Nusbaumer^1^, Lucas Marques da Cunha^1^, Claus Wedekind^*^

Department of Ecology & Evolution, University of Lausanne, Lausanne, Switzerland

^*^correspondence: Claus Wedekind, [claus.wedekind@unil.ch](mailto:claus.wedekind@unil.ch), orcid.org/0000-0001-6143-4716

^1^shared first authorship

**Table S1** (next page). Likelihood ratio tests on generalized linear mixed-effects models (GLMM) on mortality (A), and linear mixed-effects models (LMM) on hatching time (B), larval length at hatching (C), larval growth during 14 days post-hatching (D), yolk sac volume at hatching (E) and yolk consumption during 14 days post-hatching (F). Models including or lacking the term of interest were compared to the reference model to determine the significance of the effect tested. Significant p-values are highlighted with bold letters.

|  |  | *S-metolachlor* | | | | | | *diazinon* | | | | | | | |
| --- | --- | --- | --- | --- | --- | --- | --- | --- | --- | --- | --- | --- | --- | --- | --- |
| Model terms | Effect tested | AIC | d.f. | χ^2^ | | *P* | | AIC | | d.f. | | χ^2^ | | | *P* |
| 1. *Mortality* | |  |  |  | |  | |  | |  | |  | | |  |
| t+p+s+d |  | 194.2 |  |  | |  | | 176.8 | |  | |  | | |  |
| p+s+d | t | 190.8 | 2 | 0.5 | | 0.76 | | 173.7 | | 2 | | 0.9 | | | 0.64 |
| t+s+d | p | 192.2 | 1 | 0.0 | | 1.00 | | 174.8 | | 1 | | 0.0 | | | 1.00 |
| t+p+d | s | 192.2 | 1 | 0.0 | | 1.00 | | 176.1 | | 1 | | 1.4 | | | 0.24 |
| t+p+s | d | 211.0 | 1 | 18.7 | | **<0.001** | | 185.8 | | 1 | | 11.1 | | | **<0.001** |
| t+p+s+d+t\|p | t x p | 202.5 | 5 | 1.7 | | 0.89 | | 186.9 | | 5 | | 0.0 | | | 1.00 |
| t+p+s+d+t\|s | t x s | 198.4 | 5 | 5.9 | | 0.32 | | 183.4 | | 5 | | 3.4 | | | 0.65 |
| t+p+s+d+t\|d | t x d | 195.7 | 5 | 8.6 | | 0.13 | | 182.9 | | 5 | | 3.9 | | | 0.57 |
| 1. *Hatching time* | |  |  |  | |  | |  | |  | |  | | |  |
| t+p+s+d |  | 4288.0 |  |  | |  | | 4390.8 | |  | |  | | |  |
| p+s+d | t | 4335.4 | 2 | 51.4 | | **<0.001** | | 4507.4 | | 2 | | 120.6 | | | **<0.001** |
| t+s+d | p | 4287.3 | 1 | 1.3 | | 0.26 | | 4390.4 | | 1 | | 1.6 | | | 0.21 |
| t+p+d | s | 4331.2 | 1 | 45.2 | | **<0.001** | | 4439.9 | | 1 | | 51.1 | | | **<0.001** |
| t+p+s | d | 4485.7 | 1 | 199.7 | | **<0.001** | | 4390.8 | | 1 | | 150.8 | | | **<0.001** |
| t+p+s+d+t\|p | t x p | 4294.0 | 5 | 4.0 | | 0.55 | | 4399.3 | | 5 | | 1.4 | | | 0.92 |
| t+p+s+d+t\|s | t x s | 4297.1 | 5 | 1.0 | | 0.96 | | 4397.9 | | 5 | | 2.8 | | | 0.73 |
| t+p+s+d+t\|d | t x d | 4297.7 | 5 | 0.3 | | 1.00 | | 4398.7 | | 5 | | 2.1 | | | 0.84 |
| 1. *Length at hatching* | |  |  |  | |  | |  | |  | |  | | |  |
| t+p+s+d |  | 599.1 |  |  | |  | | 741.1 | |  | |  | | |  |
| p+s+d | t | 778.9 | 2 | 183.8 | | **<0.001** | | 881.7 | | 2 | | 144.6 | | | **<0.001** |
| t+s+d | p | 597.9 | 1 | 0.8 | | 0.37 | | 741.5 | | 1 | | 2.4 | | | 0.12 |
| t+p+d | s | 615.1 | 1 | 18.0 | | **<0.001** | | 758.0 | | 1 | | 18.9 | | | **<0.001** |
| t+p+s | d | 865.1 | 1 | 268.0 | | **<0.001** | | 931.2 | | 1 | | 192.1 | | | **<0.001** |
| t+p+s+d+t\|p | t x p | 605.3 | 5 | 3.8 | | 0.58 | | 749.3 | | 5 | | 1.8 | | | 0.88 |
| t+p+s+d+t\|s | t x s | 607.8 | 5 | 1.3 | | 0.93 | | 751.1 | | 5 | | 0.0 | | | 1.00 |
| t+p+s+d+t\|d | t x d | 600.8 | 5 | 8.3 | | 0.14 | | 738.0 | | 5 | | 13.1 | | | **0.02** |
| 1. *Growth* | |  |  |  | |  | |  | |  | |  | | |  |
| l+t+p+s+d |  | 605.9 |  |  | |  | | 727.8 | |  | |  | | |  |
| l+p+s+d | t | 621.7 | 2.00 | 19.8 | | **<0.001** | | 742.6 | | 2.00 | | 18.7 | | | **<0.001** |
| t+p+s+d | l | 635.5 | 1.00 | 31.4 | | **<0.001** | | 774.0 | | 1.00 | | 48.2 | | | **<0.001** |
| l+t+s+d | p | 603.9 | 1.00 | 0.0 | | 1.00 | | 725.8 | | 1.00 | | 0.0 | | | 1.00 |
| l+t+p+d | s | 605.8 | 1.00 | 1.9 | | 0.17 | | 727.9 | | 1.00 | | 2.0 | | | 0.15 |
| l+t+p+s | d | 631.0 | 1.00 | 27.1 | | **<0.001** | | 754.3 | | 1.00 | | 28.5 | | | **<0.001** |
| l+t+p+s+d+t\|p | t x p | 614.5 | 5.00 | 1.4 | | 0.92 | | 737.1 | | 5.00 | | 0.7 | | | 0.98 |
| l+t+p+s+d+t\|s | t x s | 614.1 | 5.00 | 1.8 | | 0.88 | | 735.1 | | 5.00 | | 2.7 | | | 0.74 |
| l+t+p+s+d+t\|d | t x d | 612.7 | 5.00 | 3.2 | | 0.67 | | 735.9 | | 5.00 | | 1.9 | | | 0.87 |
| 1. *Yolk volume at hatching* | |  |  |  |  | |  | |  | |  | |  |  |  |
| h+t+p+s+d |  | 6724.6 |  |  | |  | | 6891.6 | |  | |  | | |  |
| h+p+s+d | t | 6722.5 | 2 | 1.8 | | 0.40 | | 6887.9 | | 2 | | 0.3 | | | 0.84 |
| t+p+s+d | h | 6734.9 | 1 | 12.2 | | **<0.001** | | 6911.6 | | 1 | | 22.0 | | | **<0.001** |
| h+t+s+d | p | 6736.5 | 1 | 13.9 | | **<0.001** | | 6902.0 | | 1 | | 12.5 | | | **<0.001** |
| h+t+p+d | s | 6722.6 | 1 | 0.0 | | 1.00 | | 6901.1 | | 1 | | 11.5 | | | **<0.001** |
| h+t+p+s | d | 6758.0 | 1 | 35.3 | | **<0.001** | | 6903.4 | | 1 | | 13.8 | | | **<0.001** |
| h+t+p+s+d+t\|p | t x p | 6731.9 | 5 | 2.8 | | 0.74 | | 6899.2 | | 5 | | 2.3 | | | 0.80 |
| h+t+p+s+d+t\|s | t x s | 6734.6 | 5 | 0.0 | | 1.00 | | 6898.7 | | 5 | | 2.8 | | | 0.73 |
| h+t+p+s+d+t\|d | t x d | 6726.3 | 5 | 8.4 | | 0.14 | | 6887.8 | | 5 | | 13.8 | | | **0.02** |
| 1. *Yolk consumption* | | |  |  | |  | |  | |  | |  | |  |  |
| v+g+t+p+s+d |  | 3443.1 |  |  | |  | | 3544.6 | |  | |  | | |  |
| v+g+p+s+d | t | 3443.2 | 2 | 4.1 | | 0.13 | | 3551.5 | | 2 | | 10.9 | | | **0.004** |
| g+t+p+s+d | v | 4000.8 | 1 | 559.7 | | **<0.001** | | 4129.5 | | 1 | | 586.9 | | | **<0.001** |
| v+t+p+s+d | g | 3442.8 | 1 | 1.7 | | 0.19 | | 3543.8 | | 1 | | 1.2 | | | 0.27 |
| v+g+t+s+d | p | 3441.1 | 1 | 0.0 | | 1.00 | | 3542.6 | | 1 | | 0.0 | | | 1.00 |
| v+g+t+p+d | s | 3443.7 | 1 | 2.6 | | 0.10 | | 3542.6 | | 1 | | 0.0 | | | 0.95 |
| v+g+t+p+s | d | 3477.7 | 1 | 36.6 | | **<0.001** | | 3575.8 | | 1 | | 33.2 | | | **<0.001** |
| v+g+t+p+s+d+t\|p | t x p | 3453.1 | 5 | 0.0 | | 1.00 | | 3551.6 | | 5 | | 3.0 | | | 0.70 |
| v+g+t+p+s+d+t\|s | t x s | 3452.6 | 4 | 0.0 | | 1.00 | | 3551.5 | | 4 | | 1.1 | | | 0.90 |
| v+g+t+p+s+d+t\|d | t x d | 3448.2 | 5 | 4.9 | | 0.43 | | 3551.6 | | 5 | | 3.0 | | | 0.70 |

Fixed effects: t, treatment (control, low, high); h, hatching time; l, length at hatching; v, yolk volume at hatching; g, growth.

Random effects: p, population; s, sire; d, dam.

**Table S2** Maximum-likelihood estimates of variance components for (a) hatching time, (b) length at hatching, (c) larval growth, (d) yolk sac volume at hatching, and (e) yolk consumption during the first 14 days post hatching. Based on these estimates, narrow-sense heritability (*h^2^*), mean-scaled additive genetic variance (*I_A_*), and coefficients of additive genetic variation (*CV_A_*) were calculated for each of the 5 environments that was experimentally created.

|  | *V_Tot_* | *V_A_* | *V_Dam_* | *V_D_* | *V_Block_* | *V_Pop_* | *V_Res_* | *h^2^* | *I_A_* | *CV_A_* |
| --- | --- | --- | --- | --- | --- | --- | --- | --- | --- | --- |
| 1. hatching time | | | | | | | | | | |
| control | 3.33 | 0.17 | 0.40 | <0.01 | 1.22 | 0.05 | 1.62 | 0.05 | <0.0001 | 0.60 |
| S-metolachlor low | 3.38 | 0.97 | 0.70 | <0.01 | 0.74 | 0.14 | 1.55 | 0.29 | 0.0002 | 1.43 |
| S- metolachlor high | 3.30 | 0.32 | 0.34 | 0.42 | 0.99 | <0.01 | 1.78 | 0.10 | 0.0001 | 0.82 |
| diazinon low | 3.26 | 0.85 | 0.55 | 0.34 | 0.63 | <0.01 | 1.78 | 0.26 | 0.0002 | 1.33 |
| diazinon high | 3.36 | 1.07 | 0.24 | <0.01 | 0.91 | <0.01 | 1.95 | 0.32 | 0.0002 | 1.49 |
| 1. length at hatching | | | | | | | | | | |
| control | 0.22 | 0.02 | 0.11 | 0.02 | <0.01 | 0.01 | 0.08 | 0.07 | 0.0001 | 0.98 |
| S-metolachlor low | 0.18 | <0.01 | 0.04 | 0.10 | 0.03 | 0.01 | 0.08 | <0.01 | <0.0001 | <0.01 |
| S- metolachlor high | 0.19 | 0.05 | 0.08 | 0.02 | <0.01 | <0.01 | 0.09 | 0.28 | 0.0004 | 1.91 |
| diazinon low | 0.21 | <0.01 | 0.04 | 0.07 | 0.02 | 0.02 | 0.12 | <0.01 | <0.0001 | <0.01 |
| diazinon high | 0.19 | <0.01 | 0.04 | 0.14 | <0.01 | 0.01 | 0.10 | <0.01 | <0.0001 | <0.01 |
| 1. growth | | | | | | | | | | |
| control | 0.15 | 0.03 | 0.01 | <0.01 | <0.01 | <0.01 | 0.13 | 0.18 | 0.0063 | 7.95 |
| S-metolachlor low | 0.12 | <0.01 | 0.01 | <0.01 | <0.01 | <0.01 | 0.11 | <0.01 | <0.0001 | <0.01 |
| S- metolachlor high | 0.13 | <0.01 | 0.01 | 0.05 | <0.01 | <0.01 | 0.11 | <0.01 | <0.0001 | <0.01 |
| diazinon low | 0.17 | 0.02 | 0.01 | 0.02 | <0.01 | <0.01 | 0.15 | 0.14 | 0.0056 | 7.49 |
| diazinon high | 0.15 | 0.01 | 0.01 | <0.01 | <0.01 | <0.01 | 0.14 | 0.07 | 0.0025 | 5.00 |
| 1. yolk volume at hatching | | | | | | | | | | |
| control | 132.1 | <0.01 | 8.7 | <0.01 | 11.5 | 11.1 | 100.8 | <0.01 | <0.0001 | <0.01 |
| S-metolachlor low | 147.7 | <0.01 | 34.9 | <0.01 | 2.7 | 9.6 | 100.6 | <0.01 | <0.0001 | <0.01 |
| S- metolachlor high | 121.2 | <0.01 | 7.5 | 2.3 | 11.9 | 4.1 | 97.1 | <0.01 | <0.0001 | <0.01 |
| diazinon low | 134.1 | <0.01 | 7.7 | <0.01 | 20.5 | 5.5 | 100.5 | <0.01 | <0.0001 | <0.01 |
| diazinon high | 137.5 | 54.6 | 22.2 | 15.0 | <0.01 | 6.2 | 91.7 | 0.40 | 0.0308 | 17.5 |
| 1. yolk consumption | | | | | | | | | | |
| control | 95.8 | <0.01 | 7.9 | <0.01 | 11.2 | 9.9 | 66.9 | <0.01 | <0.0001 | <0.01 |
| S-metolachlor low | 105.3 | <0.01 | 37.8 | 5.2 | <0.01 | 1.7 | 64.5 | <0.01 | <0.0001 | <0.01 |
| S- metolachlor high | 88.5 | <0.01 | 3.7 | 37.8 | 7.0 | 2.0 | 66.4 | <0.01 | <0.0001 | <0.01 |
| diazinon low | 91.8 | 9.7 | 3.6 | <0.01 | 4.5 | 3.4 | 77.9 | 0.11 | 0.0347 | 18.6 |
| diazinon high | 83.8 | 15.9 | 9.7 | <0.01 | 0.9 | 1.8 | 67.4 | 0.19 | 0.0555 | 23.6 |

V_Tot_, total variance; V_A_, additive genetic; V_D_, dominance; V_Dam_, maternal; V_Block_ : block; V_Pop_: population; V_Res_, residual

**Figure S1.** Effects of exposure to two concentrations of S-metolachlor and diazinon on (A) mortality, (B) yolk volume at hatching, and (C) yolk consumption during 14 days after hatching. The panels show means based on family means and 95% CI. See Table 1 for statistics.

**Figure S2** Relationship between female body length and (A) their clutch size and (B) mean egg size. Regression lines are drawn with 95% confidence intervals. See text for statistics.

**
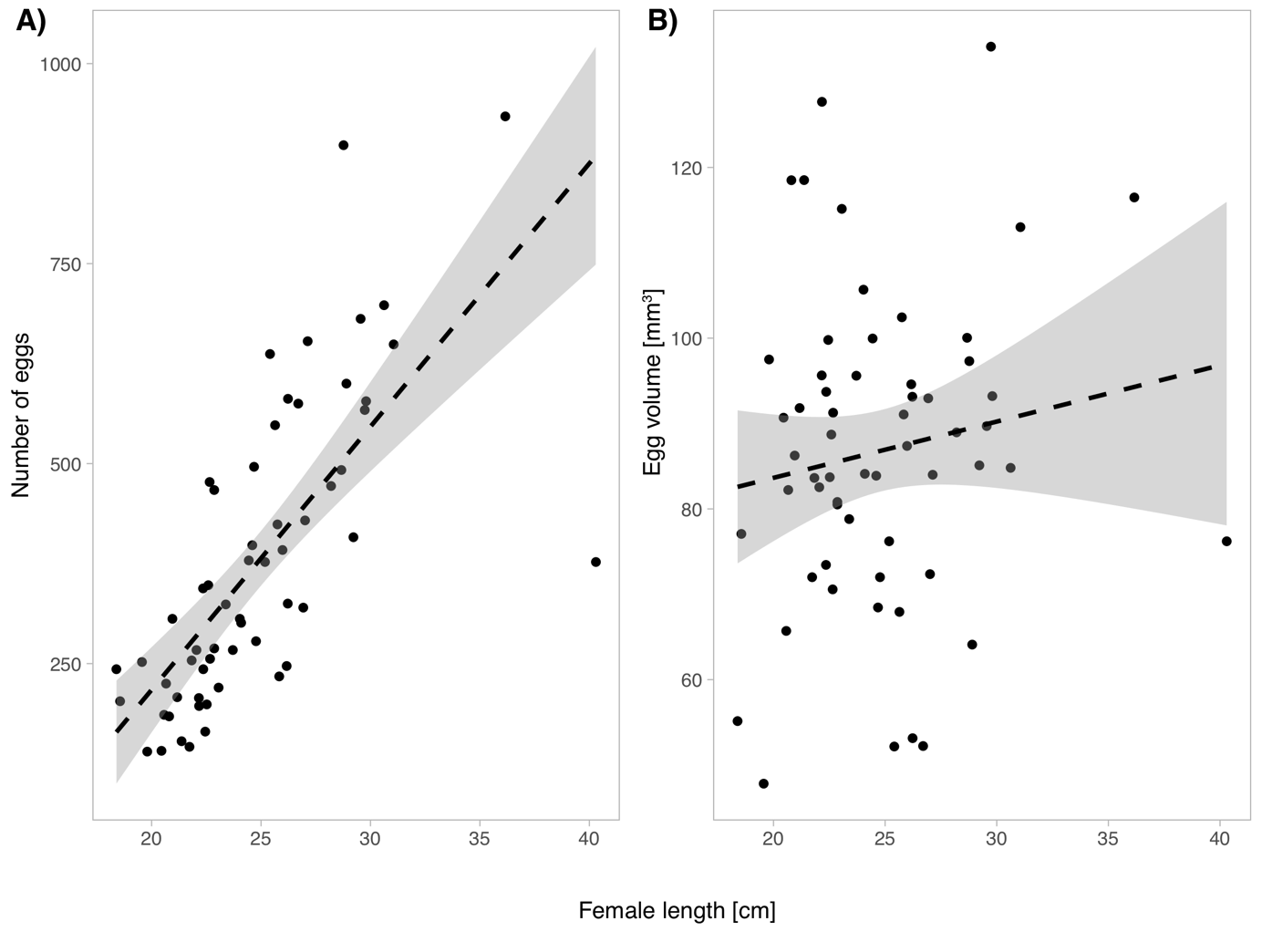
**

**Figure S3** Variation of (A) egg yellowness and (B) egg redness among the studied populations. Tukey outlier box plots with quartiles, whiskers, and outliers based on means per female. See text for statistics.

**
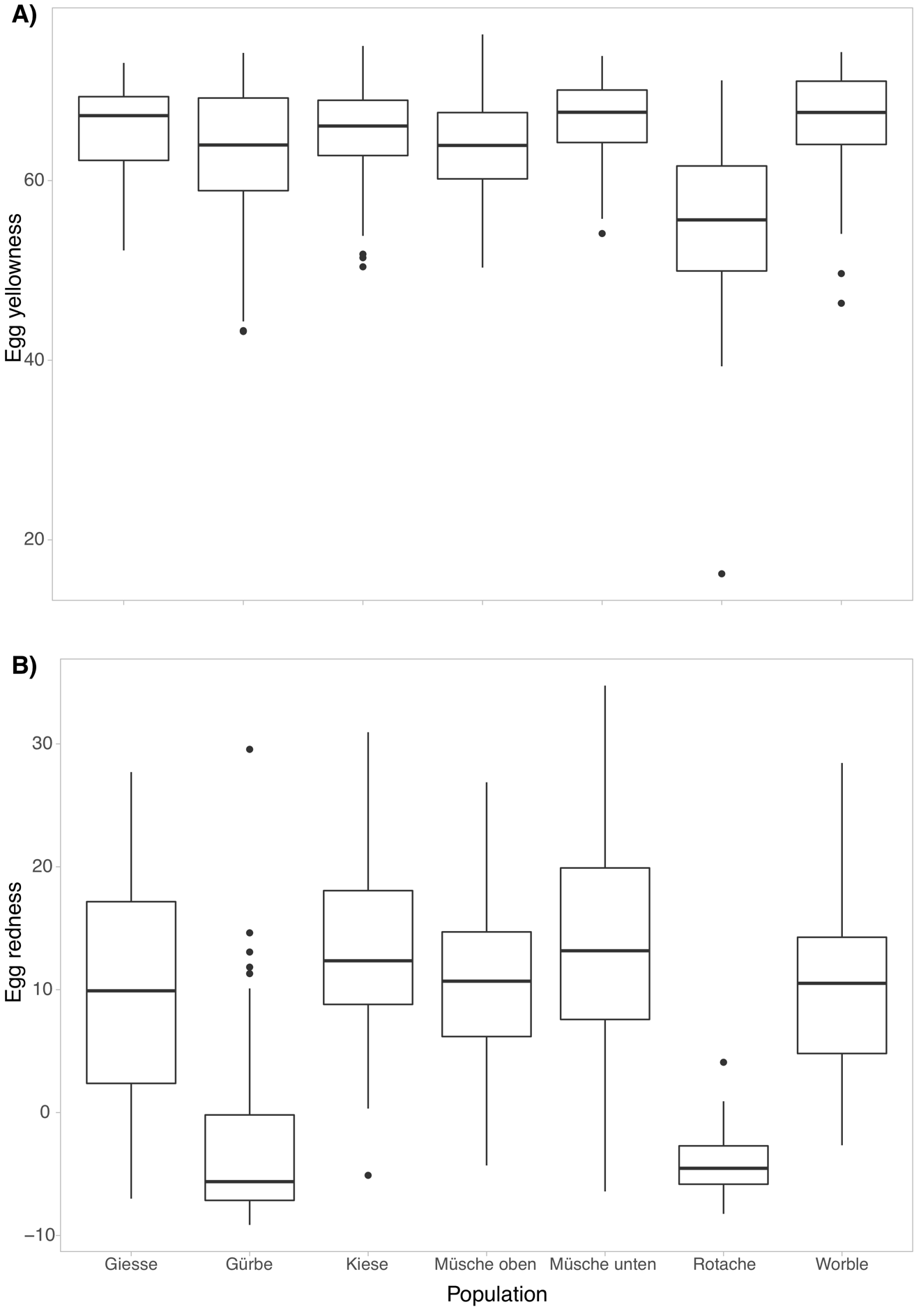
**

**Figure S4.** The reduction of hatchling length in function of egg size after exposure to (A) S-metolachlor (r = -0.27, p = 0.04) or (B) diazinon (r = -0.36, p = 0.006). Relative effects are calculated as the difference in mean length at hatching (per female) between exposed and untreated larvae, divided by the mean larval length (per female) of untreated larvae times one hundred. Regression lines are drawn with 95% confidence intervals. (Note that the analogous Figures 3A and 3B show instead model estimates.)

**Figure S5.** Reaction norms to the presence or absence (‘e’ in Table 1) of s-metolachlor (A) and diazinon (B) of paternal half-sib families for traits for which a significant exposure effect was found. There was no significant additive genetic variance for tolerance for either pesticide (e × sire interactions in Table 1).
